# Inducible transgenic expression of tripeptidyl peptidase 1 in a mouse model of late-infantile neuronal ceroid lipofuscinosis

**DOI:** 10.1371/journal.pone.0192286

**Published:** 2018-02-06

**Authors:** Yuliya Nemtsova, Jennifer A. Wiseman, Mukarram El-Banna, Peter Lobel, David E. Sleat

**Affiliations:** 1 Center for Advanced Biotechnology and Medicine, Rutgers University, Piscataway, New Jersey, United States of America; 2 Department of Biochemistry and Molecular Biology, Robert Wood Johnson Medical School, Rutgers Biomedical Health Sciences, Rutgers University, Piscataway, New Jersey, United States of America; Medical College of Wisconsin, UNITED STATES

## Abstract

Late-infantile neuronal ceroid lipofuscinosis is a fatal neurodegenerative disease of children caused by mutations resulting in loss of activity of the lysosomal protease, tripeptidyl peptidase 1 (TPP1). While *Tpp1*-targeted mouse models of LINCL exist, the goal of this study was to create a transgenic mouse with inducible TPP1 to benchmark treatment approaches, evaluate efficacy of treatment at different stages of disease, and to provide insights into the pathobiology of disease. A construct containing a loxP-flanked stop cassette inserted between the chicken-actin promoter and a sequence encoding murine TPP1 (Tg^*LSL-TPP1*^) was integrated into the ROSA26 locus in mice by homologous recombination. Tested in both transfected CHO cells and in transgenic mice, the Tg^*LSL-TPP1*^ did not express TPP1 until cre-mediated removal of the LSL cassette, which resulted in supraphysiological levels of TPP1 activity. We tested four cre/ERT2 transgenes to allow tamoxifen-inducible removal of the LSL cassette and subsequent TPP1 expression at any stage of disease. However, two of the cre/ERT2 driver transgenes had significant cre activity in the absence of tamoxifen, while cre-mediated recombination could not be induced by tamoxifen by two others. These results highlight potential problems with the use of cre/ERT2 transgenes in applications that are sensitive to low levels of basal cre expression. However, the germline-recombined mouse transgenic that constitutively overexpresses TPP1 will allow long-term evaluation of overexposure to the enzyme and in cell culture, the inducible transgene may be a useful tool in biomarker discovery projects.

## Introduction

The neuronal ceroid lipofuscinoses (NCLs) are a group of genetically distinct but clinically related hereditary lysosomal storage diseases [1]. One of the most frequently encountered [2] of the NCLs is the classical late-infantile form (LINCL, also called CLN2) which is caused by mutations in the gene encoding the lysosomal protease tripeptidyl peptidase I (TPP1) [3]. Onset of LINCL is marked by seizures and/or visual problems that become progressively more severe, eventually accompanied by dementia and loss of locomotor function. In most patients, symptoms are evident at ~3 years and progression is relentless, with death occurring between 10 and 15 years of age.

There are several *Tpp1*-targeted mouse models for LINCL that accurately recapitulate the human disease with locomotor deficits and a reduced lifespan [4–6]. Studies conducted in the original LINCL mouse model [4] have established proof-of-principle for both gene and enzyme replacement therapy but important questions regarding the reversibility of disease and the efficacy of treatment at different stages of disease remain. This is particularly important now that a treatment for LINCL has been approved [7].

In gene therapy studies on the LINCL mouse [8, 9], the timing of treatment was critical, being considerably less effective if initiated after animals become symptomatic but before there is evidence of neuronal loss. This could indicate either triggering of pathophysiological pathways that cannot be reversed by restoration of TPP1, or accumulated storage material in a subset of cell types that becomes refractory to clearance. We have also conducted enzyme replacement therapy (ERT) in the LINCL mouse [10, 11] and again found that earlier treatment was again more effective. However, an unexpected finding was that a subset of highly-symptomatic animals responded well to treatment, with significantly increased lifespan [10]. Remarkably, loss of locomotor function appeared to be partially reversible in the subset of animals that responded well.

Thus, while there are promising data, it remains unclear whether treatment of highly-symptomatic LINCL will result in positive results. In part, this may reflect limitations of therapeutic distribution in older, symptomatic mice–for example, distribution of gene therapy vectors may be broader in the small brains of neonatal animals, where best outcomes have been observed. For ERT, existing storage may impede transcytosis and distribution of the recombinant TPP1 throughout the brain. Uncorrected peripheral disease may also play a role.

With these considerations in mind, we decided to create a transgenic mouse model with inducible TPP1. This would allow constitutive or organ-specific restoration of TPP1 at any desired stage of disease for evaluation of efficacy without distribution of therapeutic as a potential confounding variable. In addition, as a best-case scenario for treatment, this model would also provide a benchmark to judge efficacy of therapies that restore TPP1 activity. Our approach was to use a tamoxifen-inducible cre/ERT2 system; however, we encountered inherent limitations that precluded creation of an inducible mouse model with the desired characteristics. In this report, we outline these problems as a case study for others attempting similar experiments.

## Methods and materials

### Animals

Animals were maintained and used following protocols approved by the Rutgers University and Robert Wood Johnson Medical School Institutional Animal Care and Use Committee which specifically approved this study (“Preclinical evaluation of therapy in an animal model for LINCL,” protocol I09-0274-4). Euthanasia was conducted using an intraperitoneal administration of a 1:3 dilution of Euthasol (Delmarva Labs) containing 130 mg pentobarbital sodium (barbituric acid derivative) and 16.7 mg phenytoin sodium. Survival studies were conducted using humane endpoints in compliance with Rutgers IACUC policies (https://orra.rutgers.edu/sites/orra.rutgers.edu/files/IACUC_Files/Rutgers%20IACUC%20Policy%20Handbook.pdf). Animals were inspected daily and euthanized if moribund as defined by a body condition score of 1 (IACUC policy #6) or paralysis. Euthanasia occurred one hour or less after animals were identified as moribund. Survival studies were conducted for up to 700 days, at which point surviving animals were euthanized. In analyzing survival data, moribund animals that were euthanized were not censored and were considered equivalent to animals that died naturally. In total, the study used 1074 mice, of which 128 were used in experimental studies and 946 were used to establish the desired genetic models. One hundred and seventy-seven animals died naturally, primarily from LINCL disease before onset of morbidity, 788 were euthanized including 6 moribund animals, and 99 were alive at the time of manuscript submission. Where noted, we also include historical controls from over ten years of maintaining C57BL/6 *Tpp1* mouse models. *Tpp1*^*-/-*^ mice show signs of disease progression but death typically occurs suddenly (possibly from disease-related seizures) when feeding and grooming behaviors remained normal and before they become moribund as defined above. However, if animals appeared ataxic but not moribund, food was placed on the bottom of the cage to facilitate feeding. All animal workers were trained to treat the animals carefully, thus minimizing stress that could also result in fatal seizures. Both male and female animals were used for the study as we have detected no gender-specific effects in life-span or other phenotypes of the LINCL mouse model (S1 Fig).

**Fig 1:**
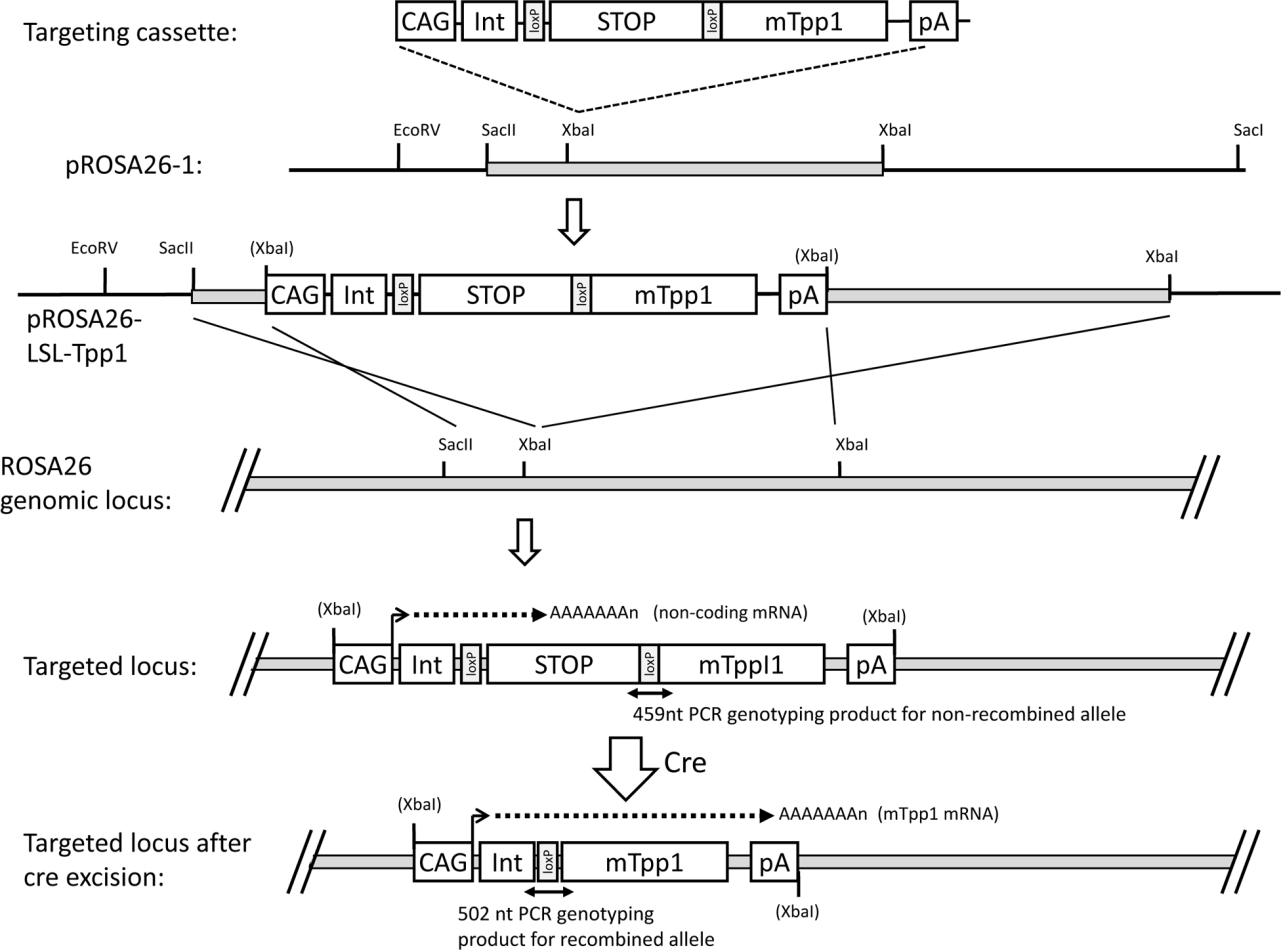
Targeting strategy for LSL-TPP1 transgene. CAG, chicken-actin promoter; Int, chicken actin splice donor, rabbit globin splice acceptor intron; STOP, stop cassette composed of PGK-neo / 4 x poly(A) sites / RNA polymerase pause site / linker containing stop codons in all reading frames; mTpp1, mouse TPP1 coding sequence; pA, rabbit globin poly(A) site.

*Tpp1*^*-/-*^ mice were in a C57BL/6 background and genotyped for the targeted *Tpp1* gene as described previously [4]. Genotyping the Tg^*LSL-TPP1*^ transgene was conducted using the primer sets depicted in Fig 1: before cre-recombination, Forward CCTAGAGCGGCCTGAATAGTTA, Reverse GCAAAAGTGAGACTCAGCTCTT, product size 459 nts; after recombination, Forward GCAAAGAATTCGGTACCTGGTTA, Reverse CCCTACATAGCGATGAAACTCAG, product size 502 nts. Genotyping PCR reactions for both recombined and unrecombined LSL-TPP1 contained a 324 nucleotide internal positive control (forward, CTAGGCCACAGAATTGAAAGATCT; reverse, GTAGGTGGAAATTCTAGCATCATCC). Mice expressing cre/ERT2 transgenes were obtained from The Jackson Laboratory (Bar Harbor, Maine) and were genotyped using their recommended protocols and primers.

### DNA construct

Plasmid pCAG-Cre was obtained from Addgene (Cambridge, MA). The Cre insert was removed by digestion with EcoRI and NotI and replaced with an EcoRI/NotI digested PCR fragment containing the entire coding sequence of the murine TPP1 cDNA (S2 Fig). This PCR fragment was engineered to introduce KpnI and SacI sites between the upstream EcoRI cloning site and the 5’-end of the TPP1 open reading frame. The KpnI and SacI sites were used to insert a PCR a loxP-flanked neo and stop cassette (LSL) derived from plasmid PGK-neo 4xSV40-pA RNA-PPS [12] kindly provided by Dr. Dieter Saur (S3 Fig). The entire inducible expression construct was amplified using NheI-flanked primers, and the PCR product digested with NheI and inserted into the XbaI site of pROSA26.1. Two positive clones were identified by restriction mapping and sequences verified by sequencing. Primer sequences for cloning and sequencing are available upon request.

### Generation of transgenic mouse

Mice containing the LSL-TPP1 transgene in the ROSA26 locus are referred to as Tg^*LSL-TPP1*^ and were generated by the Transgenic/Knockout Mouse Shared Resource of The Cancer Institute of New Jersey. The pROSA26-derived construct was linearized with XhoI prior to electroporation into the 129/B6 F1 hybrid ES cell line v6.5 [13]. Preliminary screening of ES cells for correct 5’-integration of Tg^*LSL-TPP1*^ into the ROSA26 locus was conducted by Southern blotting using a cloned PCR-amplified region of ROSA26 corresponding to nucleotides 113076032 to 113077227 of *Mus musculus* strain C57BL/6J chromosome 6 (GRCm38.p4) (S3 Fig). HindIII digestion of wild-type or correctly integrated ES cell DNA results in fragments detectable by Southern blotting of 4396 and 7355 nts, respectively. Twenty-two potential positive clones were identified from 310 screened. Correct integration at both the 5’ and 3’ sites of insertion was subsequently verified by sequence analysis of long-range PCR products spanning these junctions (S4 Fig). Two positive clones were karyotyped and used for microinjection into C57BL/6J blastocytes that were implanted into pseudopregnant Swiss-Webster females using standard techniques. A chimeric male was subsequently bred with C57BL/6J female. Animals were used after at least one backcross against C57BL/6J and thus were in a predominantly (≥75%) C57BL/6J but mixed genetic background. The transgene was maintained in a hemizygous state.

### Tamoxifen induction

Tamoxifen (Sigma T5648) was dissolved in corn oil containing 2% ethanol at a concentration of 20mg/ml by shaking at 37°C overnight and was stored in the dark at 4°C. Six-week old and 5 day-old mice were treated with an intraperitoneal dose of 200 mg tamoxifen / kg body weight. For 6-week animals, the dose was administered every 24 hours for 5 days and for 5-day animals, the dose was administered twice in one day with a 4 hr interval between doses. Six-week old animals were housed individually during and after treatment to prevent tamoxifen cross contamination due to grooming or coprophagy [14].

### TPP1 assay

TPP1 was measured using an endpoint assay with Ala-Ala-Phe-AMC substrate as described previously [15].

### Statistics

Statistical analyses were conducted using Graphpad Prism 7 for Windows, version 7.04.

## Results and discussion

### Rationale for design of the inducible TPP1 transgene

Design of the LSL-TPP1 transgene construct is shown in Fig 1. In the original LINCL model mouse, *Tpp1* is inactivated by the synergistic effects of two different defects: an Arg446His missense mutation, which is equivalent to a late-onset human allele, and insertion of a neomycin selection cassette within an adjacent intron, resulting in a disruption of normal splicing [4]. This allele is designated as *Tpp1*^-^. The neomycin cassette is loxP-flanked and germline expression of cre removes this cassette, generating the *Tpp1*^f^ allele [6]. The *Tpp1*^f^ allele produces normal levels of properly spliced transcript, albeit with the Arg446His mutation. While *Tpp1*^-/-^ mice have <0.2% wild type TPP1 activity and a median lifespan of ~4 months, *Tpp1*^f/f^ animals have ~6% wild type TPP1 activity and a median lifespan of ~20 months [6]. Given that very low levels of TPP1 can have this profound effect on disease progression, a major consideration for the design of the inducible transgene was to absolutely minimize any expression of TPP1 in the absence of induction. In a previous study, the effectiveness of different loxP-flanked transcriptional stop elements was assayed by their insertion between a strong promoter and a reporter cassette [12]. The most efficient stop cassette consisted of the resistance marker PGK-neo followed by four polyadenylation sites upstream of a human RNA polymerase pause site with linker containing stop codons in all reading frames. We therefore chose this stop cassette and inserted it with flanking loxP sites upstream of the murine TPP1 coding sequence (S2 and S3 Figs). This was inserted downstream of the strong CAG promoter and the cassette was transferred into pROSA26-1 to allow targeted recombination when generating the transgenic animals.

### Testing the LSL-TPP1 construct *in vitro*

**Fig 2:**
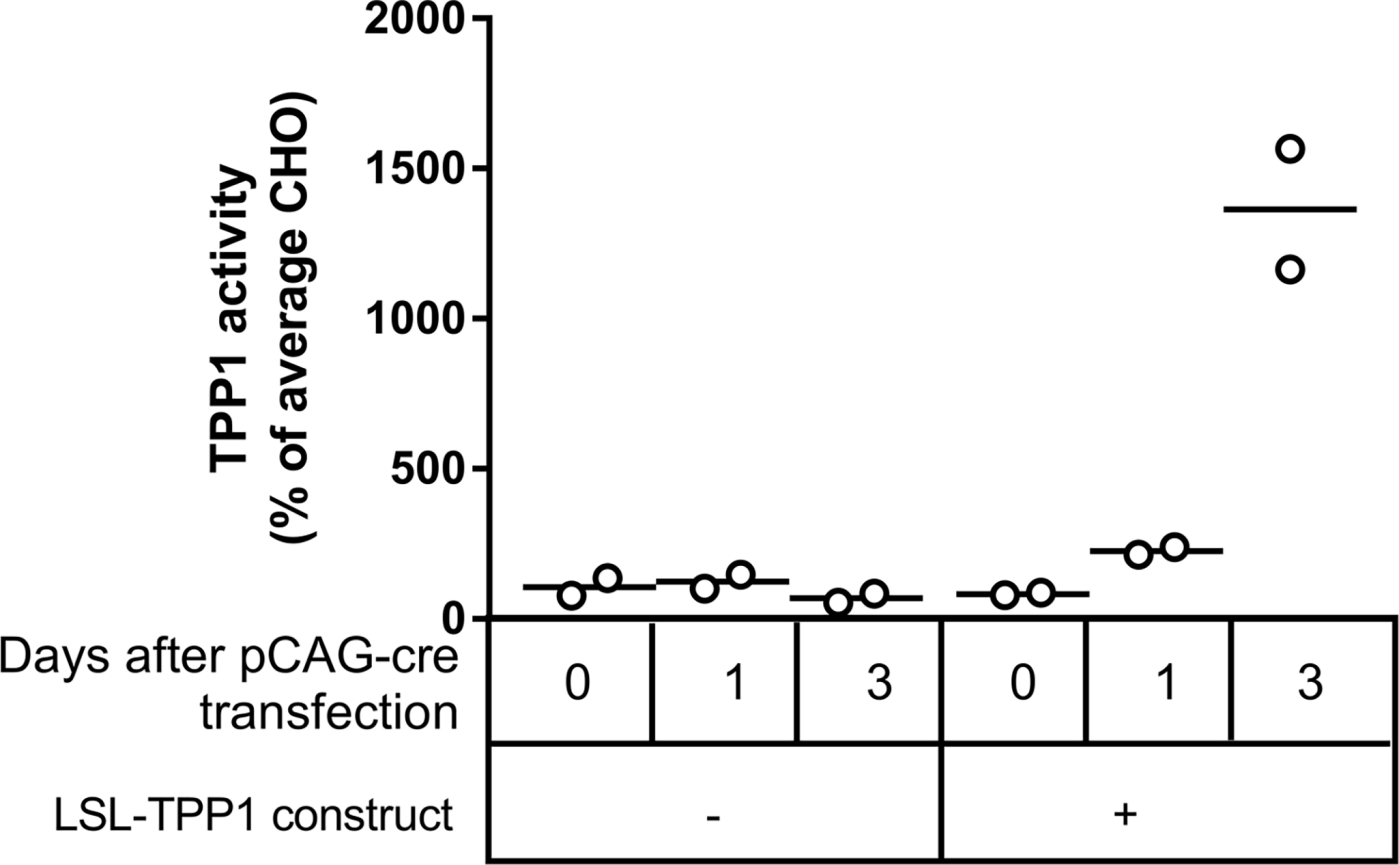
Testing LSL-TPP1 in CHO cells. CHO cells were stably transfected with an AhdI fragment of pROSA26-Tg^*LSL-TPP1*^ that contained only the CAG-LSL-TPP1 sequence. After selection with neo, CHO controls or cells containing CAG-LSL-TPP1 were transiently transfected with pCAG-cre and TPP1 activity measured at t = 0, 1 and 3 days. TPP1 activities were normalized to protein concentration and expressed as percentage of average CHO cell activity in the absence of CAG-LSL-TPP1. TPP1 activities in cells transfected with the transgene at 3 days were significantly elevated compared to all other conditions (p values ranging from 0.0003–0.0006 using Tukey’s multiple comparison test). All other comparisons had p values >0.7642.

**Fig 3:**
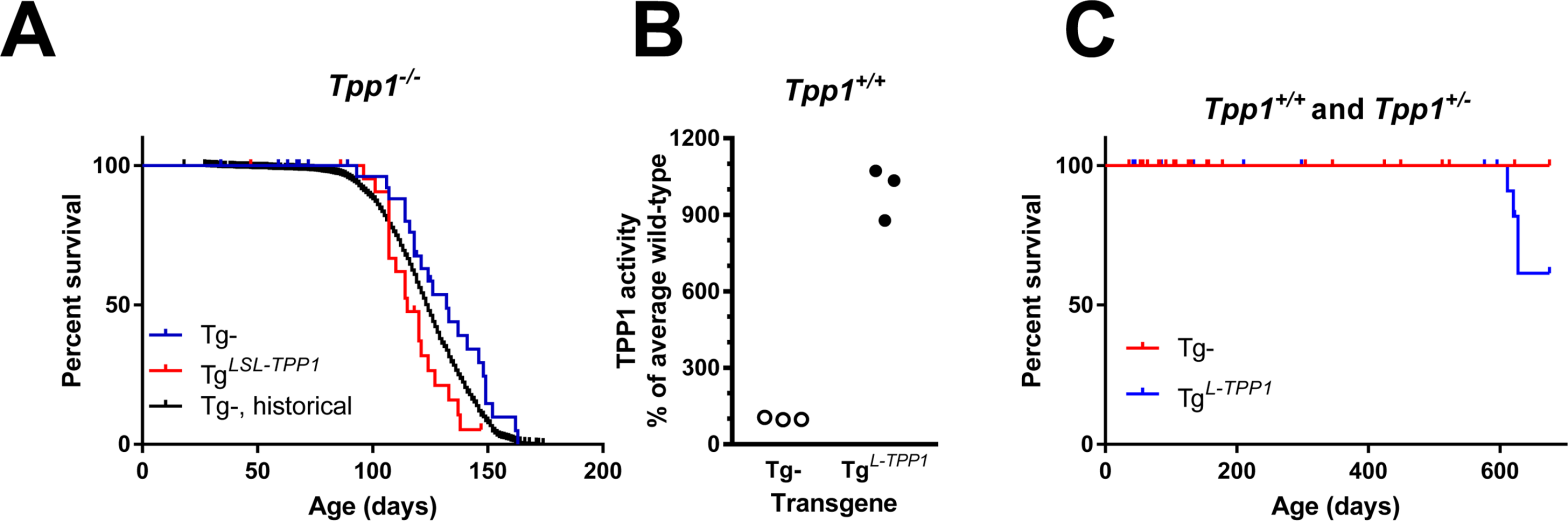
Testing LSL-TPP1 in mice. Where indicated, the stop element was constitutively removed from Tg^*LSL-TPP1*^ mice by crossing with female animals containing the Zp3-cre transgene. A. Survival of animals expressing Tg^*LSL-TPP1*^ before cre-mediated recombination (n = 56) or after (Tg^*L-TPP1*^) (n = 28). *Tpp1*^*-/-*^ (n = 1560) and *Tpp1*^*+/+*^ (n = 394) animals without transgene are historical controls. The ratio of median survival for animals with Tg^*LSL-TPP1*^ (115 days) or controls without the transgene (132 days) was 0.8712 (95% CI 0.4716 to 1.61). B. Brain TPP1 activity in wild-type animals containing Tg^*L-TPP1*^ (n = 3 animals per genotype). P value for significance between TPP1 activity in animals with or without transgene calculated using an unpaired two-tailed t-test was 0.0001. C. Survival of animals expressing Tg^*L-TPP1*^ or littermates lacking the transgene in a *Tpp1*^*+/+*^ or *Tpp1*^*+/-*^ background. Note that median survivals were undefined.

Although we sequenced the entire LSL-TPP1 portion of the final targeting construct, we also decided to verify the function of the transgene *in vitro* before proceeding with mouse transgenesis. The transgene plasmid was linearized with AhdI to excise the CAG-LSL-TPP1 sequence which was stably transfected into CHO cells using neo selection (Fig 2). We then transiently transfected pCAG-cre and measured TPP1 activity at timed intervals after transfection. Transfection with pCAG-cre had no effect on CHO cells lacking LSL-TPP1. In contrast, transfection of cells containing LSL-TPP1 with pCAG-cre resulted in a time-dependent increase in TPP1 activity, with levels three days after transfection being ~14-fold higher than average CHO. These data indicated that: 1) transfection with the LSL-TPP1 construct did not result in detectable increases in TPP1 activity in the absence of cre-recombination; 2) the stop element was correctly removed by cre-recombination as planned (Fig 1); 3) the mouse TPP1 open reading frame was highly expressed upon removal of the stop element.

### Testing the LSL-TPP1 construct *in vivo*

A mouse was created with CAG-LSL-TPP1 integrated into the ROSA26 locus, with the transgene designated as Tg^*LSL-TPP1*^. Our primary concern with this approach was the potential for leakiness of the transgene due to incomplete transcriptional suppression by the LSL cassette, which likely would be reflected by increased survival. However, the lifespan of *Tpp1*^*-/-*^ animals containing Tg^*LSL-TPP1*^ was very similar to *Tpp1*^*-/-*^ animals without the transgene, indicating that the LSL cassette was effectively preventing the expression of transgenic TPP1 as intended (Fig 3A). To ensure that transgenic TPP1 was expressed *in vivo* after cre-mediated excision of the LSL cassette, we induced constitutive recombination by crossing male Tg^*LSL-TPP1*^ mice with female Zp3-cre mice (The Jackson Laboratory, Bar Harbor, Maine), a transgenic C57BL/6J line that expresses cre within the oocyte from the zona pellucida three gene. Removal of the LSL cassette from Tg^*LSL-TPP1*^ to create the recombined transgene, designated Tg^*L-TPP1*^, resulted in a ~10-fold increase in TPP1 activity in brain compared to *Tpp1*^*+/+*^ animals lacking the transgene (Fig 3B), consistent with the CHO cell transfection studies. This again confirmed that cre-mediated recombination could occur as planned and that the TPP1 transgene was functional after removal of the LSL cassette. Initial survival analysis indicated that there appears to be no acute toxicity from chronic TPP1 overexpression (Fig 3C).

### Initial recombination studies in *Tpp1*^*-/-*^ mice

**Fig 4:**
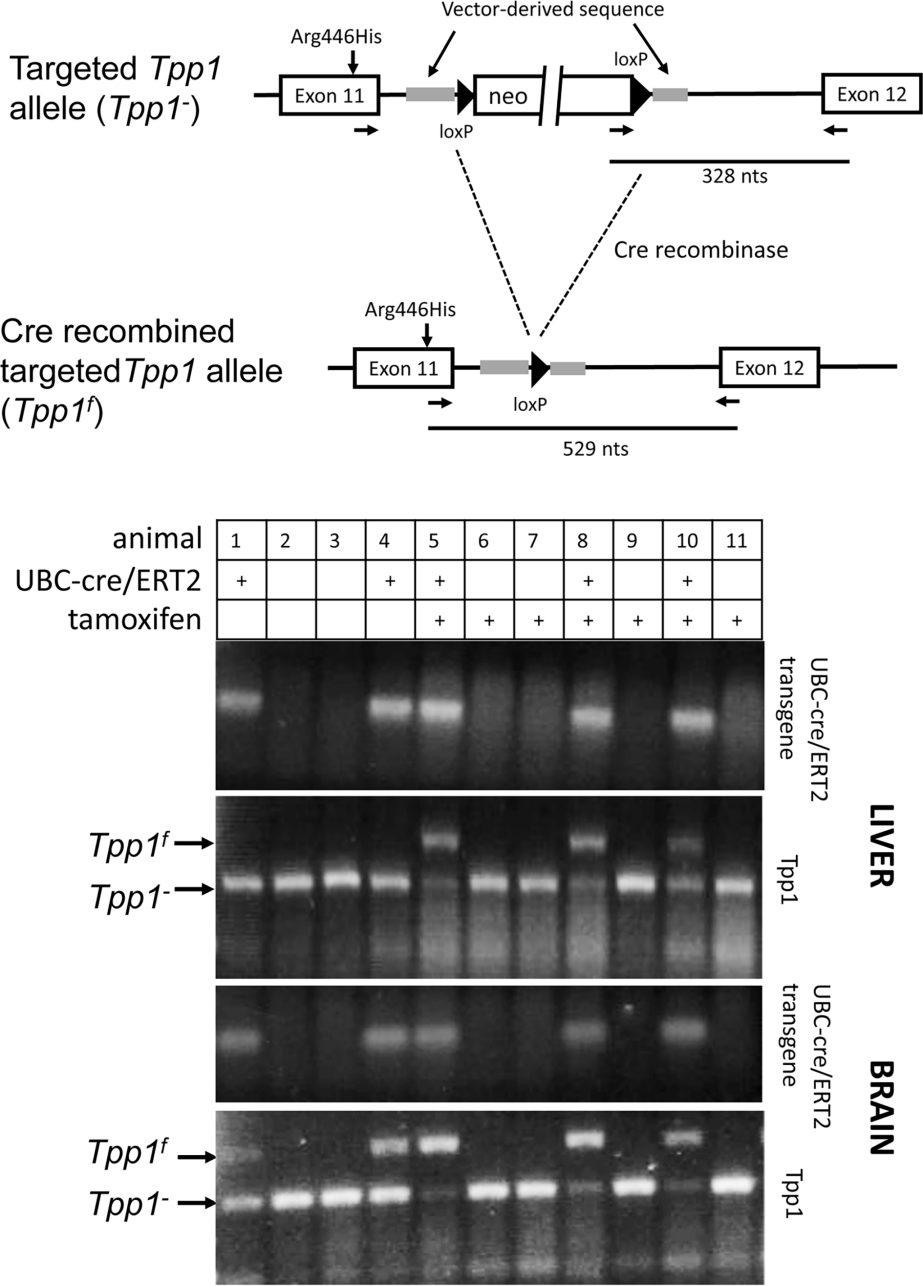
Cre recombination in *Tpp1*^*-/-*^ transgenic mice containing Tg^*UBC-cre/ERT2*^.

**Fig 5:**
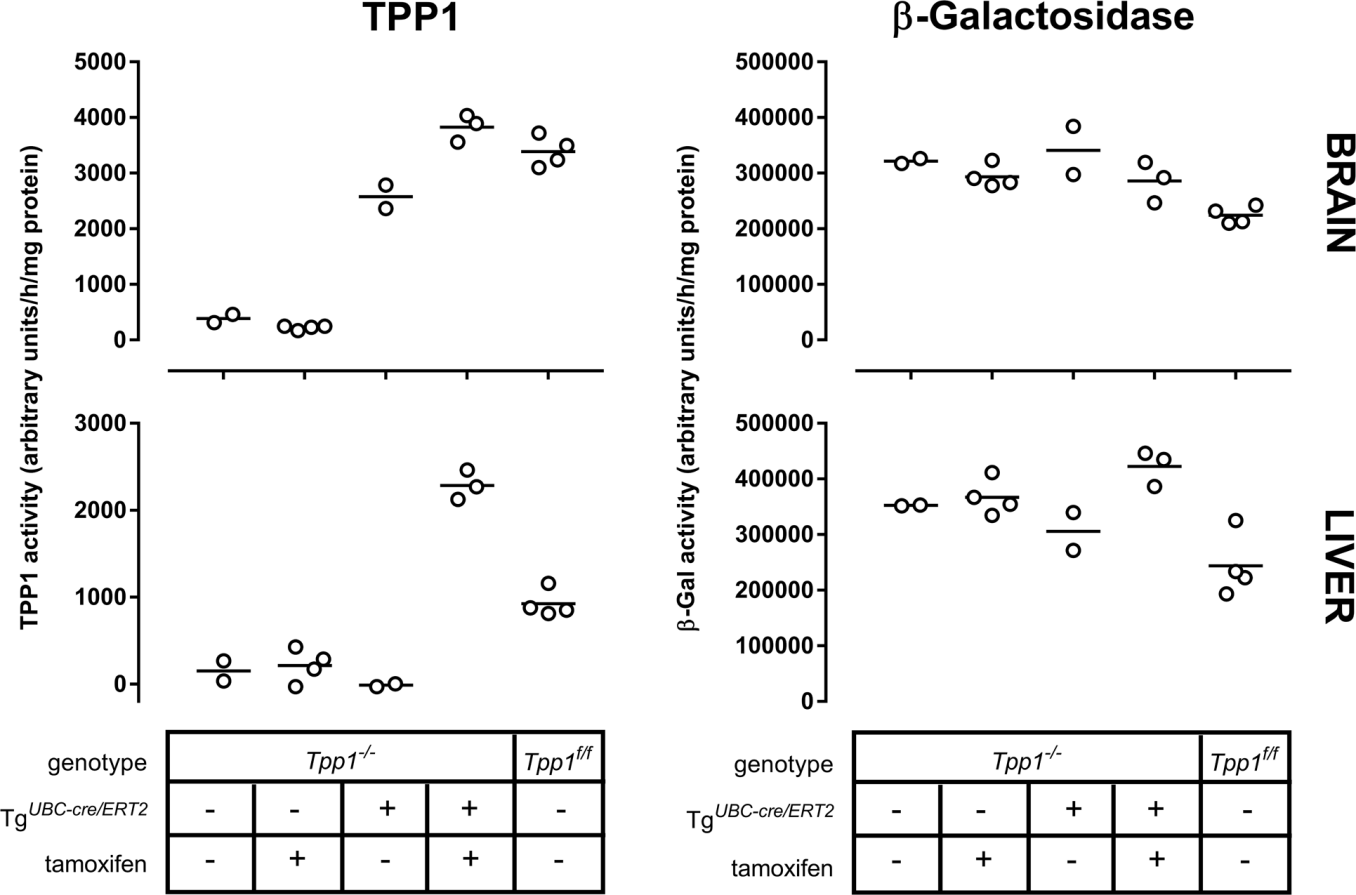
TPP1 activity in *Tpp1*^*-/-*^ transgenic mice containing Tg^*UBC-cre/ERT2*^ after tamoxifen-induced cre-recombination. TPP1 activity in *Tpp1*^*-/-*^ transgenic mice containing Tg^*UBC-cre/ERT2*^ without tamoxifen treatment was compared with other cohorts by one way ANOVA with Tukey’s multiple comparisons test. In brain, there was no significant difference in TPP1 activity between *Tpp1*^*-/-*^ animals with or without treatment, and no significant difference between *Tpp1*^*f/f*^ and tamoxifen-treated *Tpp1*^*-/-*^ animals with Tg^*UBC-cre/ERT2*^. All other comparisons were significant with p values ranging from <0.0001 to 0.0088. β-Galactosidase activity was significantly lower in *Tpp1*^*f/f*^ animals compared to *Tpp1*^*-/-*^ animals with or without treatment and untreated *Tpp1*^*-/-*^ animals with Tg^*UBC-cre/ERT2*^ (p values ranging from 0.0065 to 0.0449) while there was no significant difference between other comparisons. In liver, there was no significant difference in TPP1 activity between untreated *Tpp1*^*-/-*^ animals, treated *Tpp1*^*-/-*^ animals and untreated *Tpp1*^*-/-*^ animals with Tg^*UBC-cre/ERT2*^. All other comparisons were significant with p values ranging from <0.0001 to 0.0021. β-Galactosidase activity was significantly lower in *Tpp1*^*f/f*^ animals compared to *Tpp1*^*-/-*^ animals without treatment and tamoxifen-treated *Tpp1*^*-/-*^ animals with Tg^*UBC-cre/ERT2*^ (p values ranging from 0.0016 to 0.0125) while there was no significant difference between other comparisons.

Prior to initiating induction studies using animals containing Tg^*LSL-TPP1*^ we decided to benchmark our approach using the *Tpp1*^*-/-*^ mice, where cre-mediated removal of the neo selection marker to generate the *Tpp1*^f^ allele allows for normal splicing of the transcript containing the Arg446His mutation, resulting in *Tpp1*^f/f^ animals having ~6% of wild-type levels of TPP1 activity [6]. A transgenic line that allows for constitutive inducible cre expression, B6.Cg-Tg(UBC-cre/ERT2)1Ejb/2J (referred to here as Tg^*UBC-cre/ERT2*^) [16] was crossed with *Tpp1*^*-/-*^ mice and we conducted tamoxifen induction experiments at 6 weeks of age (Figs 4 and 5). PCR genotyping results are shown in Fig 4.

Animals were genotyped for Tg^*UBC-cre/ERT2*^ and the two different *Tpp1* alleles (*Tpp1*^-^ and *Tpp1*^f^) in brain and liver. Amplification from the unrecombined *Tpp1*^*-*^ allele results in a 328 nt product, while the cre-recombined *Tpp1*^*f*^ allele results in a 528 nt product. In the absence of the cre/ERT2 transgene, there was no detectable recombination of the *Tpp1*^*-*^ allele, without (animals 2 and 3) or with (animals 6,7,9 and 11) tamoxifen treatment. In animals treated with tamoxifen in the presence of Tg^*UBC-cre/ERT2*^, we detected efficient cre recombination as shown by detection of the *Tpp1*^*f*^ allele. While this PCR assay was not designed to be quantitative, the efficiency of tamoxifen-induced cre-mediated excision appeared to be consistent with earlier studies using this cre-driver [16] and appeared to be most efficient in brain.

Two control mice (Fig 4, animals 1 and 4) contained both the LSL-TPP1 and cre-driver transgenes but were not treated with tamoxifen. In liver, we detected no recombination of the *Tpp1*^*-*^ allele in these animals. In brain, there was a minor but clearly detectable PCR product in the absence of tamoxifen treatment that corresponded to the recombined allele, *Tpp1*^*f*^. This raised concerns that the Tg^*UBC-cre/ERT2*^ may have basal functional recombinase activity in the absence of a tamoxifen in brain. Even with this apparent constitutive activity, recombination was greatly enhanced by tamoxifen (Fig 4, compare relative intensities of *Tpp1*^-^ and *Tpp1*^f^ PCR products in animals 5, 8 and 10 versus 1 and 4), indicating that the effector was entering the brain and triggering cre-mediated recombination as anticipated.

Cre-mediated conversion of *Tpp1*^-^ to *Tpp1*^f^ should result in increased TPP1 expression and as expected, elevated TPP1 activity was detected in the tamoxifen-treated *Tpp1*^-/-^ animals containing Tg^*UBC-cre/ERT2*^ (Fig 5). In brain, the induced TPP1 activity was similar to that of *Tpp1*^*f/f*^ animals. In liver, induced activity was greater than measured in *Tpp1*^*f/f*^ animals. This was unexpected and the underlying explanation is unclear.

TPP1 activities were also measured in the control mice that contained both the LSL-TPP1 and cre-driver transgenes but which were not treated with tamoxifen. In liver, there was no increase in TPP1 activity compared to *Tpp1*^-/-^ animals indicating that, as expected, there was no cre-mediated recombination of the LSL-TPP1 transgene. In brain, TPP1 activity was elevated indicating cre-mediated recombination in the absence of tamoxifen.

**Fig 6:**
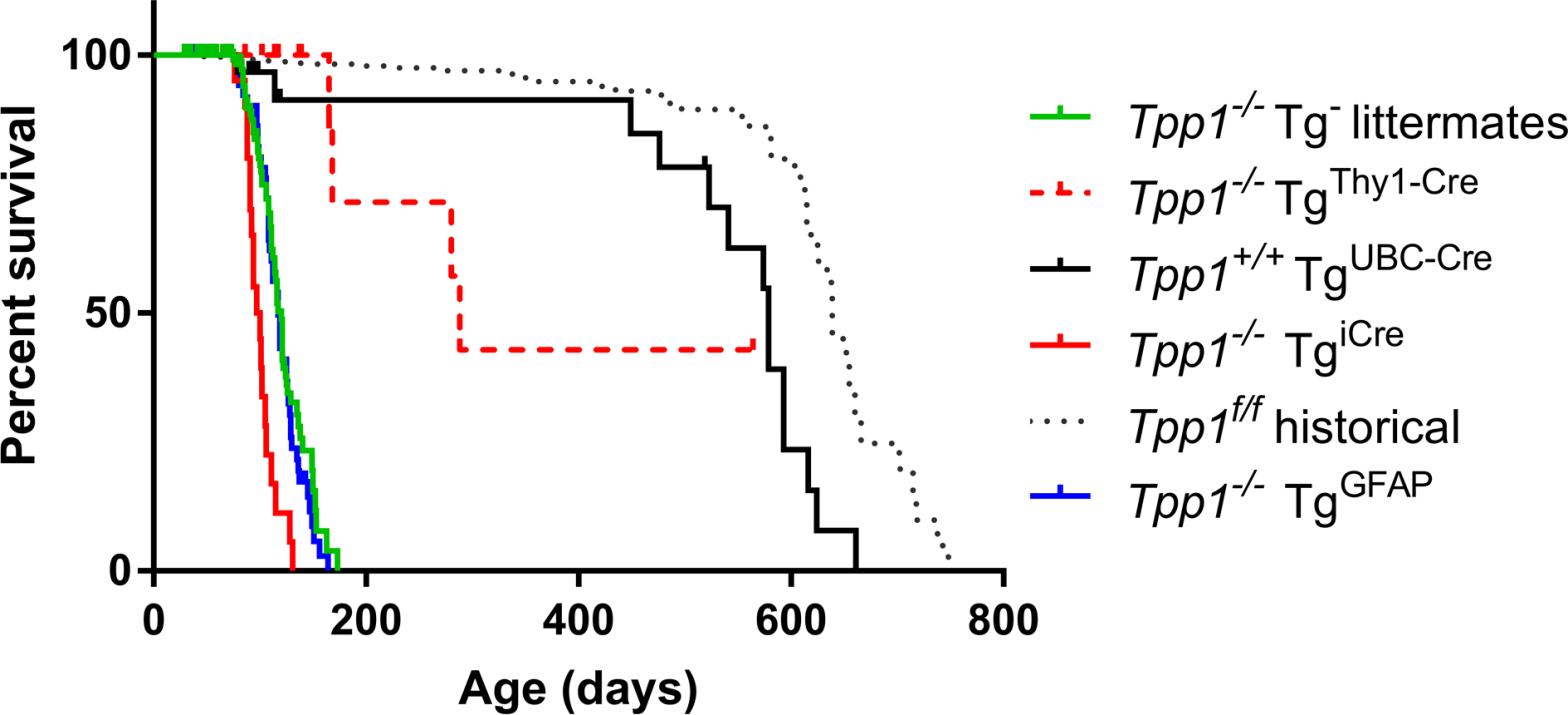
Survival of *Tpp1*^*-/-*^ mice in the presence of cre/ERT2 transgenes. Note that animals were not treated with tamoxifen. Median survival and median survival ratio compared to *Tpp1*^*-/-*^ littermate controls for each strain was: *Tpp1*^*-/-*^ littermate controls (n = 161, 60 deaths), median survival 124 days; *Tpp1*^*-/-*^ Tg^*UBC-cre/ERT2*^ (n = 57, 14 deaths), median survival is 579 days, ratio to *Tpp1*^*-/-*^ is 4.825 (95% CI 2.697 to 8.633); *Tpp1*^*-/-*^ Tg^*GFAP-cre/ERT2*^ (n = 54, 46 deaths), median survival is 117 days, ratio to *Tpp1*^*-/-*^ is 0.975 (95% CI 0.664 to1.432); *Tpp1*^*-/-*^ Tg^*Thy1-cre/ERT2*^ (n = 19, 4 deaths), median survival is 288 days, ratio to *Tpp1*^*-/-*^ is 2.4 (95% CI 0.8723 to 6.604); *Tpp1*^*-/-*^ Tg^*icre/ERT2*^ (n = 20, 19 deaths), median survival is 99 days, ratio to *Tpp1*^*-/-*^ is 0.8208 (95% CI 0.49 to 1.375); *Tpp*^*f/f*^ (n = 754, 50 deaths), median survival is 639 days, ratio to *Tpp1*^*-/-*^ is 5.325 (95% CI 3.659 to7.75).

Genotyping and TPP1 assay data were consistent in demonstrating basal TPP1 activity in brain due to cre activity from Tg^*UBC-cre/ERT2*^ in the absence of tamoxifen. The effect of basal TPP1 activity in brain on survival of the *Tpp1*^*-/-*^ animals is shown in Fig 6. Median survival was increased (579 days) compared to *Tpp1*^*-/-*^ littermate controls lacking the cre-driver transgene (124 days) and was comparable to the 639 day median survival of *Tpp1*^*f/f*^ animals.

### Other cre/ERT2 transgenes

Studies with the Tg^*UBC-cre/ERT2*^ transgene clearly indicated that due to the presence of significant tamoxifen-independent TPP1 activity in brain, this cre driver could not be used to create the desired inducible TPP1 model. We therefore investigated several other cre/ERT2 transgenes, using survival of the *Tpp1*^*-/-*^ animals as a primary test for tamoxifen-independent induction of TPP1 expression.

Mouse strain Tg(Thy1-cre/ERT2-EYFP)HGfng/PyngJ contains a transgene (referred here as Tg^*Thy1-cre/ERT2*^) with cre/ERT2 and enhanced yellow fluorescent protein expression driven by two separate copies of a modified mouse *Thy1* promoter region [17]. *Thy1* is expressed in neurons and other cell types and the transgene is expressed in most projection neuron populations of the central and peripheral nervous system. Compared to *Tpp1*^*-/-*^ animals, survival of the *Tpp1*^*-/-*^ animals with the Tg^*Thy1-cre/ERT*^ was increased, with a median survival of 506 days. As with the UBC-cre/ERT2 transgene, this suggested tamoxifen-independent cre recombination with Tg^*Thy1-cre/ERT*^, resulting in basal TPP1 expression in brain and possibly other tissues.

We identified two cre-driver transgenes that did not increase survival of the *Tpp1*^*-/-*^ mice. Mouse strain B6(129S4)-Et(icre/ERT2)10596Rdav/J contains a transgene (referred here as Tg^*icre/ERT2*^) that expresses a mammalian codon-optimized cre in a gene-trap construct that expresses ubiquitously throughout the brain. Strain B6.Cg-Tg(GFAP-cre/ERT2)505Fmv/J contains a transgene (referred here as Tg^*GFAP-cre/ERT2*^) that expresses cre/ERT2 driven by the glial fibrillary acidic protein (GFAP) promoter [18]. GFAP is expressed in many cell types throughout the brain including astrocytes and when this transgene is induced early in postnatal development, progeny of GFAP-expressing astroglial ancestors can differentiate into oligodendrocytes and astrocytes throughout the cerebral cortex and white matter, as well as neurons in select regions of the brain. While GFAP is not expressed to any significant degree in adult neurons, our hope was that transgenic overexpression of TPP1 via the GFAP promoter in astrocytes would allow for cross-protection of adjacent neurons through the secretion-recapture pathway [19]. Presence of either Tg^*icre/ERT2*^ or Tg^*GFAP-cre/ERT2*^ did not increase survival of *Tpp1*^-/-^ mice (Fig 6), suggesting that there was no constitutive recombination in the absence of tamoxifen that would increase basal TPP1 activity. These transgenes were used for subsequent induction studies.

**Fig 7:**
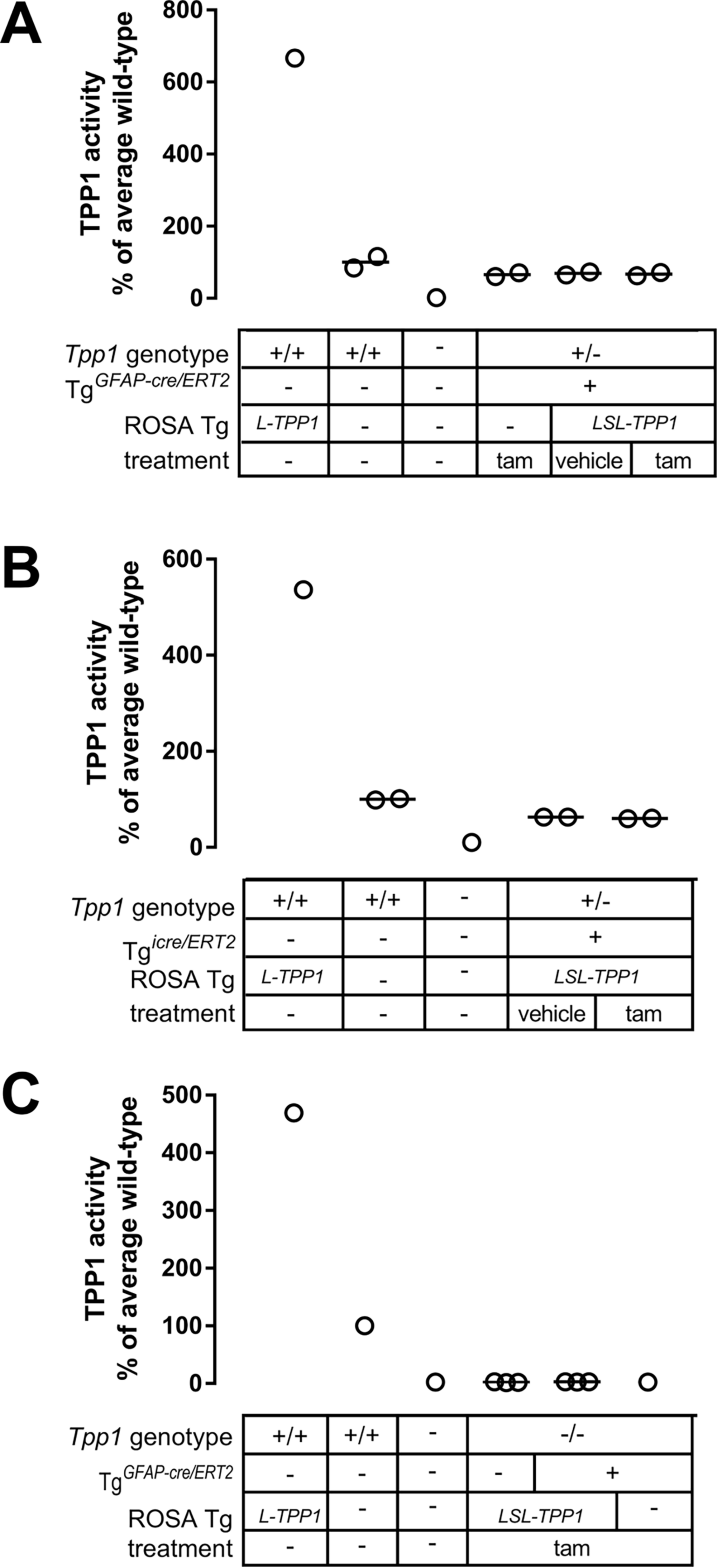
Testing tamoxifen-induced recombination of Tg^*LSL-TPP1*^ by Tg^*GFAP-cre/ERT2*^ and Tg^*icre/ERT2*^. Six-week *Tpp1*^*+/-*^ animals with indicated Tg^*LSL-TPP1*^ and Tg^*GFAP-cre/ERT2*^ (A) or Tg^*icre/ERT2*^ (B) genotypes were treated with tamoxifen as shown. Animals were killed one week after induction and assayed for TPP1 brain activity. C, 5-day old *Tpp1*^*-/-*^ animals containing Tg^*LSL-TPP1*^ with Tg^*GFAP-cre/ERT2*^ were treated as indicated, killed one month after induction, and assayed for brain TPP1 activity. Tam, tamoxifen; Other transgene is Tg^*LSL-TPP1*^ (TPP1 transgene with stop cassette) or Tg^*L-TPP1*^ (active TPP1 transgene). The mean of each experimental condition was compared with every other mean by one-way ANOVA with p values corrected using Tukey’s multiple comparison test. No significant increase in TPP1 activity was detected under any experimental condition when animals containing both Tg^*LSL-TPP1*^ and cre/ERT2 driver transgene were treated with tamoxifen compared to similar animals that were untreated or vehicle-treated.

*Tpp1*^*+/-*^ mice containing Tg^*LSL-TPP1*^ and either Tg^*GFAP-cre/ERT2*^ or Tg^*icre/ERT2*^ were treated at 6 weeks of age with tamoxifen for 5 consecutive days. One week after the final dose, animals were killed and recombination at the Tg^*LSL-TPP1*^ transgene in the brain was assayed for by PCR for the recombined allele and by TPP1 assay. *Tpp1*^*+/+*^ animals with the recombined transgene lacking the stop cassette (Tg^*L-TPP1*^) had >10-fold higher TPP1 activity compared to *Tpp1*^*+/+*^ animals lacking the transgene (Fig 3B), thus excision of the TPP1 transgene stop cassette in the context of a *Tpp1*^*+/-*^ genotype should also result in a marked increase in TPP1 activity. As expected, we detected no recombination in control animals lacking Tg^*LSL-TPP1*^ or in animals with Tg^*LSL-TPP1*^ but in the absence of tamoxifen treatment. However, as measured by PCR (Figure A in S5 Fig) or TPP1 assay (Fig 7A and 7B), we failed to detect excision of the stop cassette from Tg^*LSL-TPP1*^ when animals with either of the cre/ERT2 driver transgenes were treated with tamoxifen at 6 weeks of age. Note that, consistent with survival studies (Fig 3A), there was no detectable TPP1 activity from the unrecombined Tg^*LSL-TPP1*^ allele, demonstrating the efficacy of the stop cassette.

One possibility was that the cre transgenes were developmentally silenced so that recombinase activity could not be induced by tamoxifen at 6 weeks of age. To test this possibility, we treated neonatal *Tpp1*^*-/-*^ animals containing both Tg^*LSL-TPP1*^ and Tg^*GFAP-cre/ERT2*^ with tamoxifen. Again, we fail to detect recombination by either PCR (Figure B in S5 Fig) or TPP1 assay (Fig 7C), indicating that animal age appears not to be a critical factor for lack of inducible recombination in this model.

## Conclusions

A transgenic mouse expressing an inducible TPP1 would provide a valuable preclinical model and also a tool to shed light onto the pathobiology of LINCL. However, our efforts to develop this model were not successful with two critical technical issues encountered.

First, with two of the tamoxifen-inducible cre-driver transgenes (Tg^*UBC-cre/ERT2*^ and Tg^*Thy1-cre/ERT2*^), there was constitutive expression of cre/ERT2 in the absence of tamoxifen treatment in brain. A number of studies have reported basal levels of cre recombination to variable degrees with the Tg^*UBC-cre/ERT2*^ strain and other cre/ERT2 drivers [20–22]. In the most recent [20], widespread basal cre-recombination driven by Tg^*UBC-cre/ERT2*^ was detected in various tissues including kidney, liver, heart and lung. While we found tamoxifen-independent cre/ERT2 activity in brain, no basal activity was detected in liver. Given that very low levels of TPP1 can dramatically ameliorate the phenotype of the LINCL mouse, this level of basal cre recombination with these two transgenes was unacceptable for our purposes. More generally, our findings underscore the necessity for evaluation of the degree of tamoxifen-independent cre recombination when using cre/ERT2 driver transgenes for sensitive applications.

Second, the two other transgenes tested (Tg^*GFAP-cre/ERT2*^ or Tg^*icre/ERT2*^) failed to drive tamoxifen-inducible cre recombination *in vivo* as demonstrated directly by a failure to amplify the recombined allele and indirectly by TPP1 measurement. We do not know why these two cre driver transgenes failed to function as expected in our hands, but our results appeared to eliminate:

1. problems with formulation and/or delivery of tamoxifen to the brain
2. mutations in the TPP1 transgene or associated regulatory sequences
3. age-related epigenetic factors that prevented cre-mediated recombination, at least for the Tg^*GFAP-cre/ERT2*^

It is worth noting that efficiency of cre-recombination with Tg^*GFAP-cre/ERT2*^ was reported [18] to vary with two different reporter transgenes, with one reporter showing just 10% of the number of cre-recombined cells observed with the other. While the reasons underlying the difference in response from two different reporter constructs are not clear, it is possible that Tg^*LSL-TPP1*^ recombines very poorly for similar reasons.

While our efforts to use the cre/ERT2 system with our Tg^*LSL-TPP1*^ to create an inducible model have not been successful to date, it is possible that other cre/ERT2 driver strains with the desired characteristics exist or will be developed in the future. It is worth noting that the Tg^*LSL-TPP1*^ transgene itself performed as expected and could be useful for other applications. The mouse containing Tg^*L-TPP1*^ which was obtained by germline cre-recombination, had ~10-fold higher than physiological levels of TPP1 activity. This mouse may be useful for evaluating potential toxicities associated with long-term overexposure to TPP1, or potential benefits of TPP1 overexpression in other disease states. In addition, the ability to induce TPP1 in cultured cells containing Tg^*LSL-TPP1*^ by transfection with a cre driver plasmid would allow cell-lines to act as their own controls in studies on TPP1-dependent changes, alleviating the inherent variability encountered with cell-line to cell-line comparisons. This may facilitate cell-based biomarker discovery efforts and may help shed light on the pathobiology of LINCL.

## Supporting information

S1 Fig Untreated female and male C57BL/6 Tpp1-/- mice have similar overall survival. Median survival: female, 125 days, male, 123 days; female/male = 1.016 (95% CI 0.9241 to 1.118). Analysis based on colony records for 1761 females (1003 deaths) and 1610 males (737 deaths).
(TIF)

S2 Fig Construction of the TPP1 transgene. (TIF)

S3 Fig Construction of the TPP1 transgene (continued). (TIF)

S4 Fig Verification of transgene integration into ROSA26. (TIF)

S5 Fig PCR evaluation of cre/ERT2 mediated recombination. Animals with indicated transgenic phenotypes were treated with tamoxifen at p42 (A) or p5 (B). One month later, animals were killed and cre-mediated recombination examined by PCR screen for the recombined allele (Tg^*L-TPP1*^) with arrow indicating position of the positive PCR product. Two animals with constitutive recombination were genotyped as positive controls. DNA size marker is 100bp ladder. Gel images have been manipulated to show animals and genotypes of interest.
(TIF)
