# Supplementary figures and images for: Inducible transgenic expression of tripeptidyl peptidase 1 in a mouse model of late-infantile neuronal ceroid lipofuscinosis
Source: PLoS One. 2018 Feb 6;13(2):e0192286. doi: 10.1371/journal.pone.0192286 (PMC5800698; doi:10.1371/journal.pone.0192286)

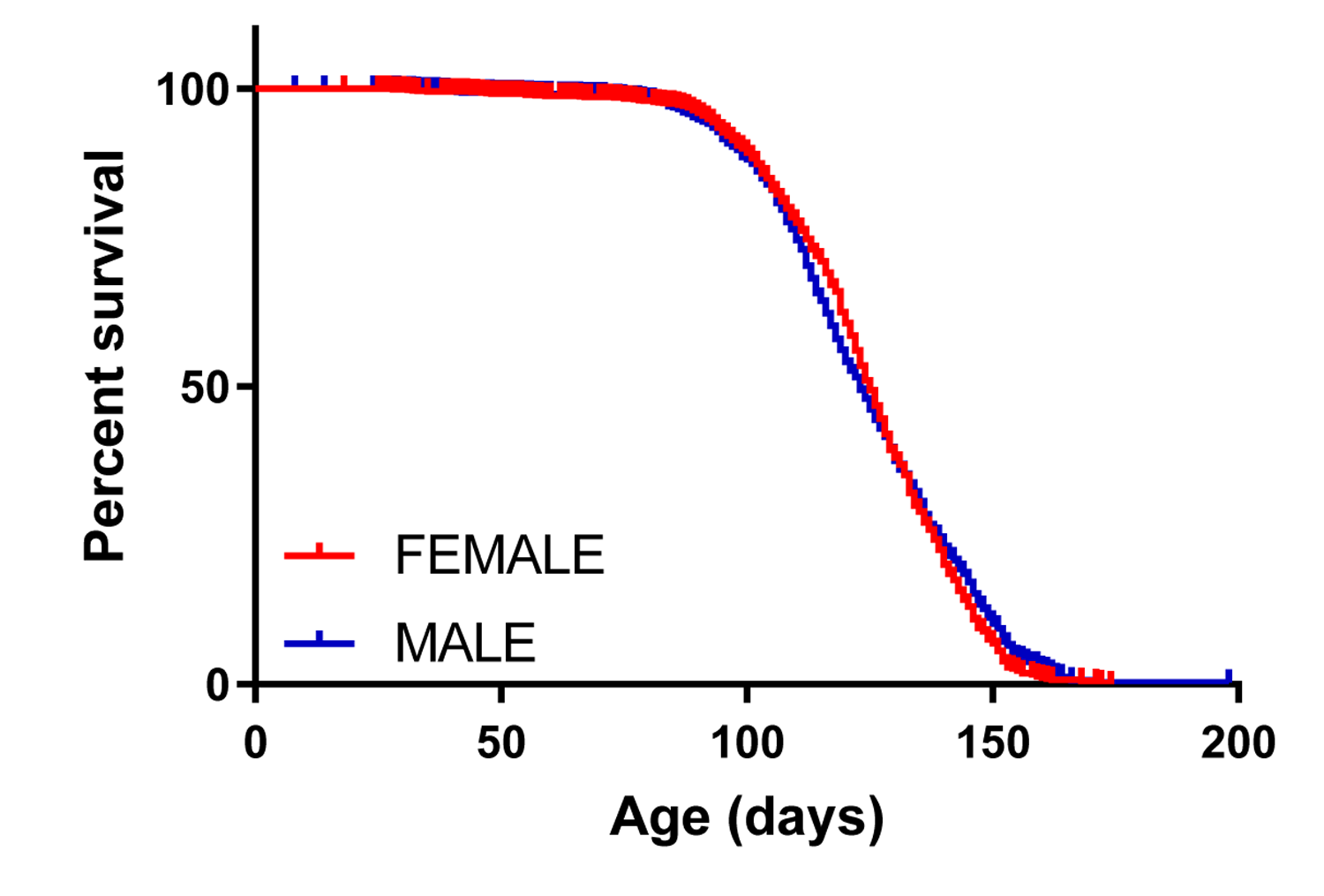

Supplement: S1 Fig — Median survival: female, 125 days, male, 123 days; female/male = 1.016 (95% CI 0.9241 to 1.118). Analysis based on colony records for 1761 females (1003 deaths) and 1610 males (737 deaths). (TIF) [file pone.0192286.s001.tif]

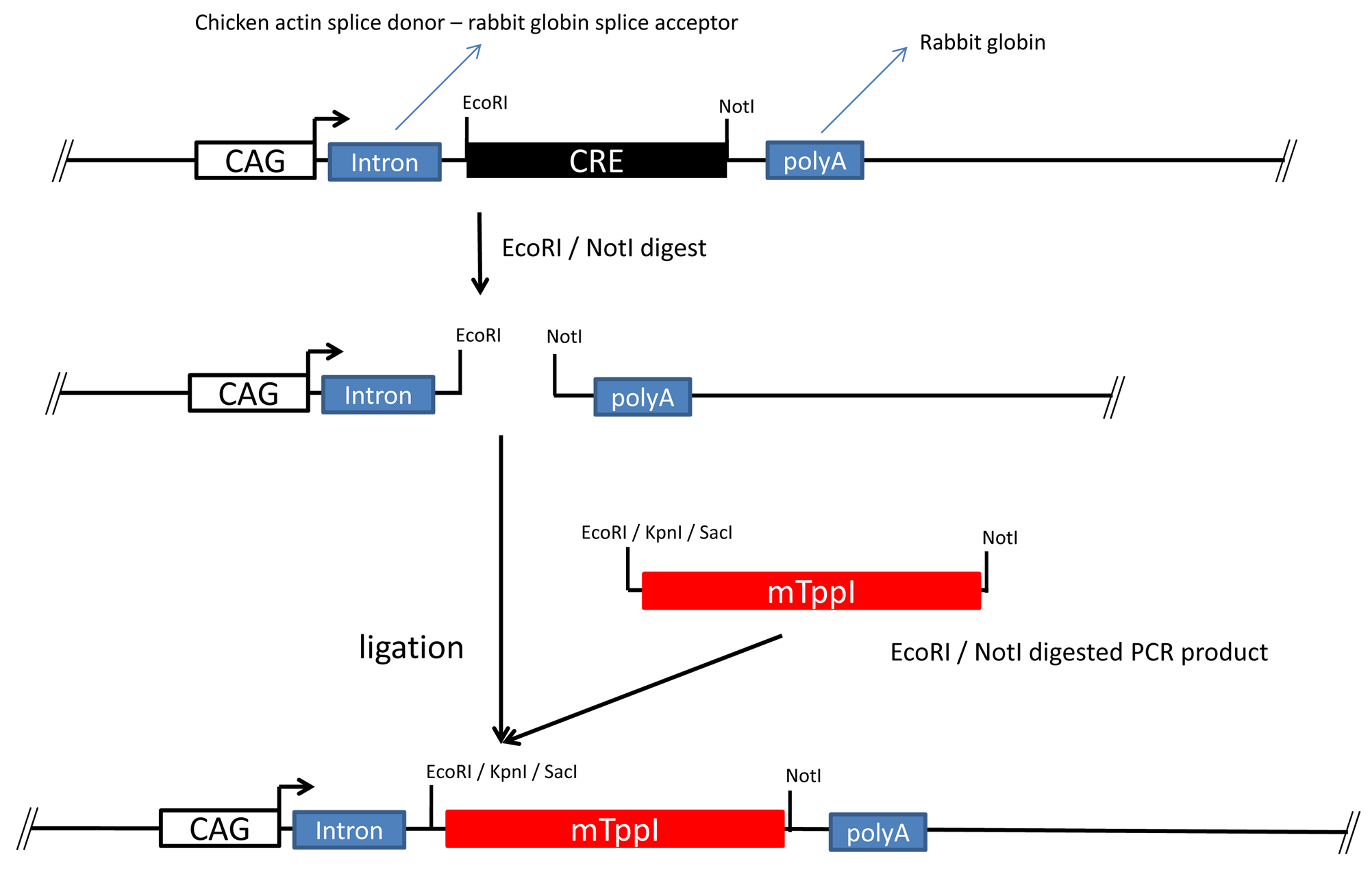

Supplement: S2 Fig — (TIF) [file pone.0192286.s002.tif]

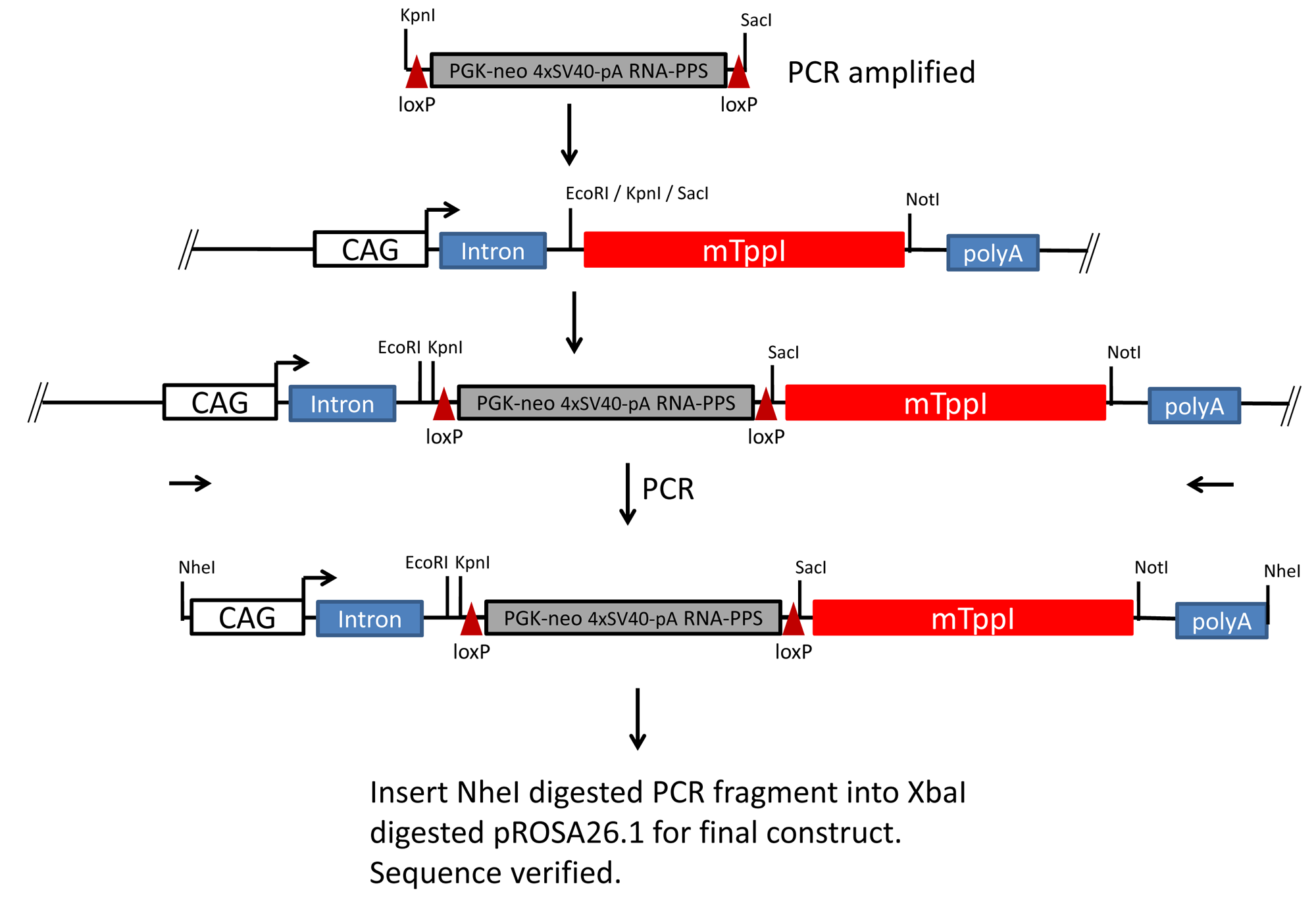

Supplement: S3 Fig — (TIF) [file pone.0192286.s003.tif]

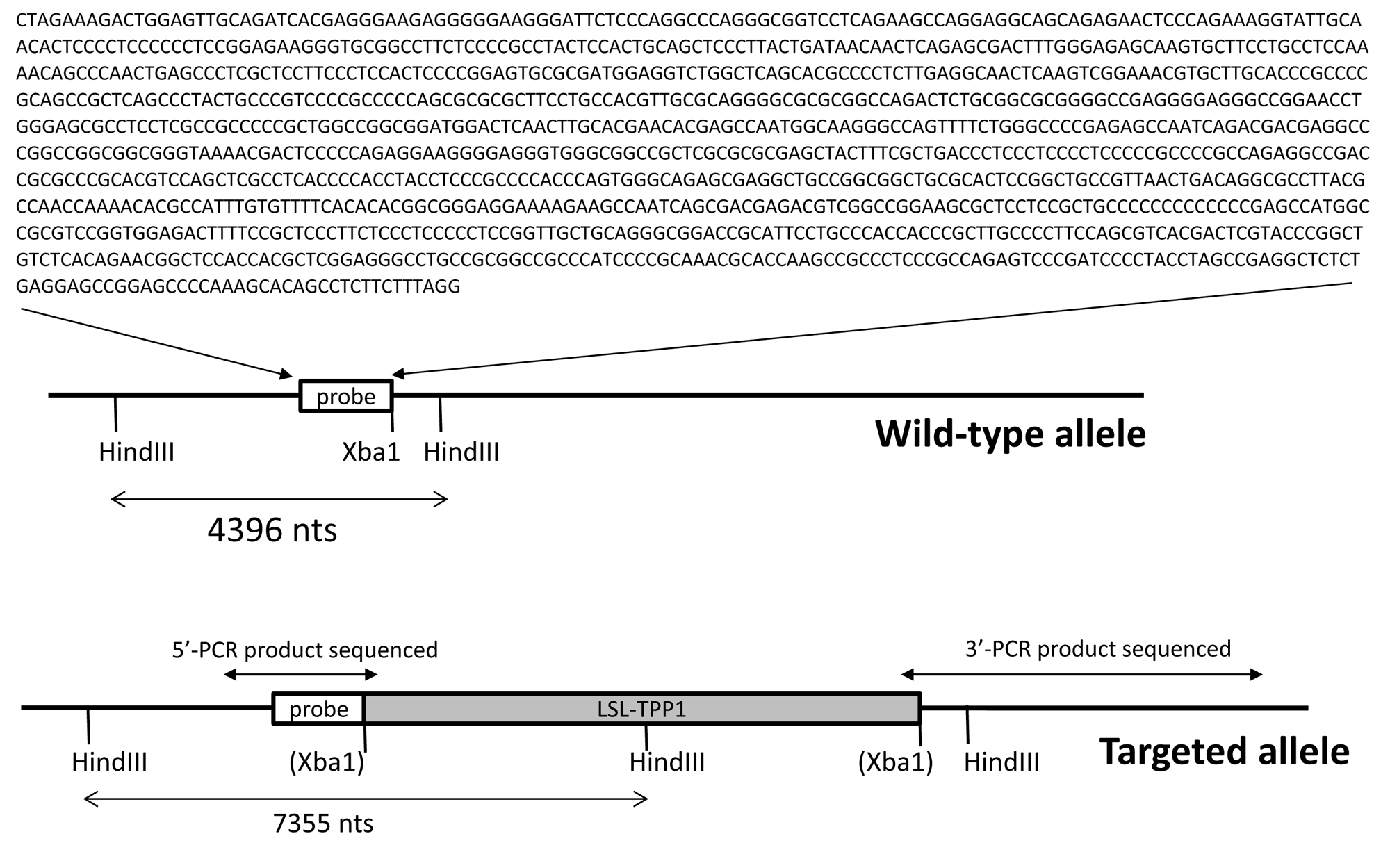

Supplement: S4 Fig — (TIF) [file pone.0192286.s004.tif]

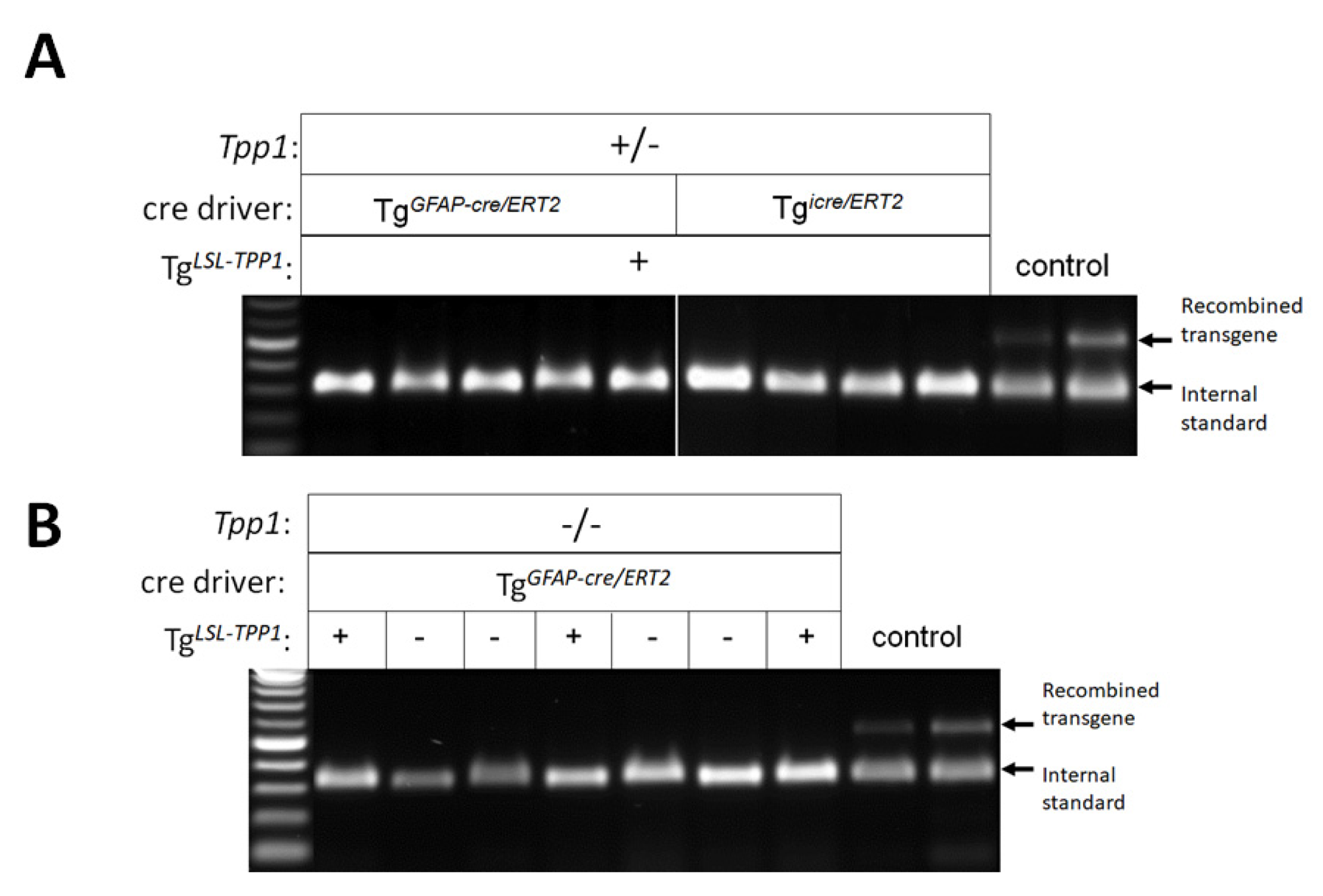

Supplement: S5 Fig — Animals with indicated transgenic phenotypes were treated with tamoxifen at p42 (A) or p5 (B). One month later, animals were killed and cre-mediated recombination examined by PCR screen for the recombined allele (TgL-TPP1) with arrow indicating position of the positive PCR product. Two animals with constitutive recombination were genotyped as positive controls. DNA size marker is 100bp ladder. Gel images have been manipulated to show animals and genotypes of interest. (TIF) [file pone.0192286.s005.tif]
